# Approaches for Health Effect Characterization in Risk-Benefit Assessment of Foods: A Comparative Case Study

**DOI:** 10.3389/fnut.2021.607929

**Published:** 2021-07-09

**Authors:** Sofie Theresa Thomsen, Maarten Nauta, Lea Sletting Jakobsen, Marianne Uhre Jakobsen, Heddie Mejborn, Malene Outzen, Morten Poulsen, Gitte Ravn-Haren, Rikke Andersen

**Affiliations:** ^1^Division of Diet, Disease Prevention and Toxicology, National Food Institute, Technical University of Denmark, Kgs. Lyngby, Denmark; ^2^Division of Risk Assessment and Nutrition, National Food Institute, Technical University of Denmark, Kgs. Lyngby, Denmark

**Keywords:** risk-benefit assessment, health effect characterization, substitution, food matrix, health impact, nutrition

## Abstract

One of the challenges in quantitative risk-benefit assessment (RBA) of foods is the choice of approach for health effect characterization to estimate the health impact of dietary changes. The purpose of health effect characterization is to describe an association between intake of a food or food component and a health effect in terms of a dose-response relationship. We assessed the impact of the choice of approach for health effect characterization in RBA in two case studies based on substitution of (i) white rice by brown rice and (ii) unprocessed red meat by vegetables. We explored this by comparing the dose-response relations linking a health effect with (i) a food component present in the food, (ii) a food based on non-specified substitution analyses, and (iii) a food based on specified substitution analyses. We found that the choice of approach for health effect characterization in RBA may largely impact the results of the health impact estimates. Conducting the calculations only for a food component may neglect potential effects of the food matrix and of the whole food on the diet-disease association. Furthermore, calculations based on associations for non-specified substitutions include underlying food substitutions without specifying these. Data on relevant specified substitutions, which could reduce this type of bias, are unfortunately rarely available. Assumptions and limitations of the health effect characterization approaches taken in RBA should be documented and discussed, and scenario analysis is encouraged when multiple options are available.

## Introduction

Quantitative risk-benefit assessment (RBA) of foods is a method used for weighing the adverse and beneficial effects of food consumption on human health, and for comparing the health impact of current consumption patterns with alternatives, such as dietary interventions (1) or changes in food cooking practices (2). Usually, a common metric such as number of deaths or summary measures of population health such as disability-adjusted life years are applied to measure risks and benefits (3, 4). RBA can be used by national and international authorities to provide support for evidence-based policy and public guidance about dietary changes. Examples include RBAs of fish consumption for specific target groups, and of consumption of nuts in the general population (5–7). Several frameworks for RBA of food have been developed under international research projects (3, 8–10). Less than a decade after these frameworks were published, a wide range of RBAs of food has been conducted (11).

RBAs can be conducted at different “levels of aggregation (9, 12).” Traditionally, three levels are identified: a food component such as vitamin D in the context of food fortification (food component level); a food product such as fish (food level); or a whole diet such as the Mediterranean diet (diet level). Most published RBAs have assessed the impact of changes in consumption at food level, assuming all other foods remain constant. However, it is recognised that a change in consumption in one food implies a change in consumption of other foods and that specific food substitutions can be either beneficial, neutral, or harmful in relation to health (13). Therefore, the impact of substitution is increasingly considered in RBA, which adds the food substitution level as a fourth level of aggregation at which RBA can be performed (14–19).

The RBA approach resembles that of food safety risk assessment (8). In this approach, a critical step is the health effect characterization, which describes the association between intake and health effect in terms of a dose-response (DR) relationship. These relationships are often derived from existing epidemiological studies (10) and expressed as relative risks for a certain health outcome, depending on the intake. In general, the DR relation can be described at different levels of aggregation as well. DR relationships can be found for specific food components, such as omega-3 fatty acids (DHA) and neurological development (20), but also for a whole food with non-specified substitution, such as fish and fatal coronary heart disease (20), and with specified food substitution. In many cases, there is only one DR model available for a health effect considered in an RBA. However, if different DR relationships exist for the same health effect, but at different levels of aggregation, a choice has to be made between them.

In theory, if the food component in a food is assumed to be solely responsible for the health effect, application of the DR relationship for the food component would predict the same response as a DR relationship for the food. As, for example, omega-3 fatty acids (DHA and EPA) in fish have a beneficial effect on the neurodevelopment of the foetus during pregnancy, this relationship should yield the same prediction as a DR for fish and neurodevelopment. As, however, fish contains more components that may impact the neurodevelopment, fatty acids do occur in other foods in the diet, and interaction with the food matrix may play a role as well, this may not be the case in practice. Moreover, if a substitution of fish with meat is specified in the epidemiological study behind the DR relationship, the estimated health impact may again be different.

It is our hypothesis that an RBA may yield very different results, and in worst-case different conclusions, depending on which approach is taken for the health effect characterization. Therefore, this paper aims to assess how the choice for the level of aggregation at which the DR relationship is specified may impact the results of RBAs. In two case studies, we explored the impact of characterizing health effects associated with substitution of foods by either relating a given health effect with the change in intake of a food component, the change in intake of foods based on non-specified substitution, or the change in intake of foods based on specified substitution of foods, respectively. The two case studies were based on (i) substitution of white rice by brown rice associated with risk of type 2 diabetes mellitus (T2DM) and (ii) substitution of unprocessed red meat by vegetables associated with myocardial infarction (MI).

## Materials and Methods

### Substitution of White Rice by Brown Rice

We investigated the differences in predicted hazard ratios between the use of dose-response relationships based on the association measures for T2DM and cereal fiber intake (21). This was done for a non-specified substitution of brown rice (i.e., substitution of brown rice with a mix of foods in the diet), a non-specified substitution of white rice (i.e., substitution of white rice with a mix of foods in the diet), and the specified substitution of white rice by brown rice (22). Brown rice (22) and substitution of white rice by brown rice (22) was found to be associated with a statistically significantly lower risk of T2DM, while white rice consumption has been found to be associated with a statistically significantly higher risk of T2DM (22). Rice, and in particular brown rice, is a source of cereal fiber. The evidence of an association between intake of fiber (non-starch polysaccharide), and lower risk of T2DM was classified as probable by the World Health Organization (23). Supporting this, a recent meta-analysis showed that cereal fiber intake is associated with a lower risk of T2DM (21). The identified associations were described by hazard ratios (HR) and are listed in Table 1. Although rice and cereal fiber intake may be associated with other health effects, only T2DM was considered for the purpose of this study.

**Table 1:** Identified association measures at different food levels.

	**Direction of association (+/–)**	**Exposure measure**	**HR (95% CI)**	**References**
**Type 2 Diabetes Mellitus**				
Non-specified substitution of brown rice^a^	–	Serv.^*d*^ <1/month 1/month to 1/week >2/week	1 0.94 (0.90, 0.98) 0.89 (0.81, 0.97)	(22)
Non-specified substitution of white rice^a^	+	Serv.^*d*^ <1/month 1–3/month 1/week2-4/week >5/week	1 1.01 (0.94, 1.08) 1.04 (0.96, 1.12) 1.11 (1.03, 1.20) 1.17 (1.02, 1.36)	(22)
Specified substitution of white rice by brown rice^a^	–	per 50 g/day	0.84 (0.79, 0.91)	(22)
(Non-specified substitution of) cereal fiber	–	per 10 g/day	0.75 (0.65,0.86)	(21)
**Myocardial Infarction**				
Non-specified substitution of vegetables^b,c^	–	per 150 g/day	0.99 (0.97, 1.01)	(24)
Non-specified substitution of unprocessed red meat^b,c^	+	per 150 g/day	1.08 (1.02, 1.14)	(24)
Specified substitution of unprocessed red meat by vegetables^b,c^	–	per 150 g/day	0.91 (0.86, 0.98)	(24)

*The identified associations between substitution of white by brown rice and type 2 diabetes mellitus and substitution of unprocessed red meat by vegetables and myocardial infarction, the type of analysis behind the association measure, the direction of the association [increased (+) or decreased (–) risk], the exposure measure, the association measure in terms of hazard ratio (HR) and 95% confidence interval (CI), and the reference*.

a *The association measures for rice are for cooked amounts*.

b *The association measures for vegetables and unprocessed red meat are for amounts as consumed*.

c *The estimates from Würtz et al. (24) are only for women*.

d *One serving (serv.) was set to 150 g of cooked rice as in Sun et al. (22)*.

### Substitution of Unprocessed Red Meat by Vegetables

We also investigated the differences between the use of dose-response relationships based on the association measures for MI and a non-specified substitution of vegetables (i.e., substitution of vegetables with a mix of foods in the diet), a non-specified substitution of unprocessed red meat (i.e., substitution of unprocessed red meat with a mix of foods in the diet), and the specified substitution of unprocessed red meat by vegetables (24). In non-specified substitution analyses, vegetable consumption was found to be associated with a lower risk of cardiovascular disease (CVD) and the association was classified as convincing by the WHO (23). Meanwhile, evidence for an association between consumption of unprocessed red meat and subtypes of CVD is inconsistent (25–27). A recently published study investigated the association between substitution of unprocessed red meat, defined as fresh and minced beef, veal, pork and lamb, by vegetables and the risk of MI (24). Substitution of unprocessed red meat by vegetables was associated with a statistically significantly lower risk of MI. In analyses not specifying the substitution, intake of unprocessed red meat was associated with a significantly higher risk of MI, whereas intake of vegetables was not associated with a statistically significantly lower MI risk. The HRs derived by Wurtz et al. (13) are listed in Table 1.

### Dose-Response Functions

Dose-response functions were derived from the association measures listed in Table 1, assuming a HR of 1 at zero consumption/substitution and a log-linear association (28):

$$\begin{array}{l} \ln\left(HR\right)=\;\beta x \end{array}$$

where x is the intake amount and β can be estimated from the HR for a given x. β-coefficients were expressed in terms of per gram substitution and used for describing the dose-response relationship as an exponential function:

$$\begin{array}{l} HR=\;e^{\beta x} \end{array}$$

A more detailed description of how the β-coefficients were calculated is given in the Supplementary Material. We applied HR estimates from Sun et al. (22) to associate white and brown rice with T2DM risk derived from non-specified and specified substitution analyses. The reported servings of white and brown rice were converted to grams per day and plotted against their associated HR. Using the same approach, a dose-response function associating cereal fiber intake with T2DM risk was derived based on the HR estimates from the meta-analysis by The InterAct Consortium (21). The dose-response functions associating unprocessed red meat and vegetables with MI risk derived from non-specified and specified substitution analyses (24) were modeled by taking the same approach.

The dose-response functions derived for the case studies were compared in a hypothetical example in which up to 50 g/day of one food was substituted with 50 g/day of another; i.e., white rice by brown rice and unprocessed red meat by vegetables, respectively. A gram by gram substitution was specified in Sun et al. (22) and Würtz et al. (24), and thus the same substitution ratio was applied in both our case studies. Substitution was conducted for consumed amounts of cooked rice and unprocessed red meat and vegetables, respectively.

An HR was calculated for each gram hypothetical substitution based on the respective dose-response functions from the two case studies. Changes in cereal fiber intake due to the substitution was calculated using data on total fiber content in uncooked rice (0.7 g/100 white rice; 4.2 g/100 g brown rice), obtained from the Danish Food Composition Databank (29). For this purpose, cooked rice amounts were converted to uncooked amounts by dividing by a factor of 2.5 and 3.8 for white rice and brown rice, respectively (30).

In quantitative RBAs, where association measures based on specified substitution analyses are not available, the association measures derived from a combination of two non-specified substitution analyses (e.g., for brown rice and white rice) can be used to characterize the health impact associated with the substitution (e.g., of white rice by brown rice). Therefore, we also compared this approach with the dose-response functions based on the association measures derived from the specified and non-specified substitutions. It was assumed that the risk reduction through increased consumption of the substituting food acts independently from the risk reduction through decreased consumption of the substituted food, so the HRs can be multiplied. The β-coefficient for the combined dose-response function, expressed in terms of per gram substitution, was therefore found by taking the sum of the β-coefficients estimated for the non-specified substitution of the substituting food (e.g., brown rice) and the substituted food (e.g,. white rice), respectively, also both expressed in per gram substitution. A more detailed description of how the HRs for a given substitution amount were calculated is given in the Supplementary Material.

The HRs calculated per gram hypothetical substitution from the different dose-response functions in each case study were plotted against the substitution amount (0–50 g/day) in order to investigate the potential impact of choosing one over the other for the health impact estimation in RBA.

All calculations were conducted using Microsoft Office Excel and HR plots were generated using R version 3.5.1 (31).

## Results

**Figure 1:**
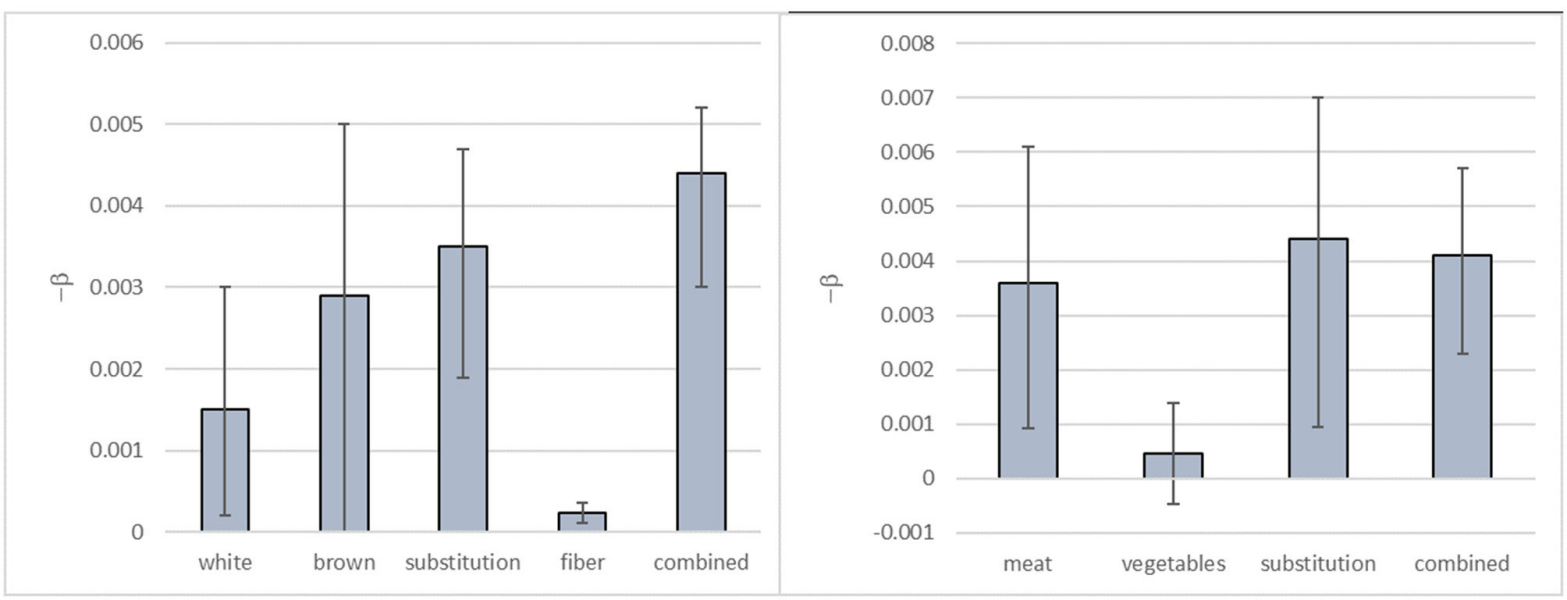
Mean estimates and 95% CI for the β-coefficients derived for log-linear dose-response functions for the substitution of white rice by brown rice and risk of T2DM (left) and the substitution of unprocessed red meat by vegetables and risk of MI (right). Note that values of –β are given. The β-coefficients are per gram increase (white rice/vegetables), per gram decrease (brown rice/unprocessed red meat) or per gram substitution (other).

The β-coefficients and the associated 95% confidence intervals (CIs) derived for the log-linear dose-response functions, based on the data shown in Table 1, are shown in Figure 1. It compares the DR relationships obtained at different levels of aggregation. Some profound differences in mean estimates of the β-coefficients are observed, but the uncertainty of the estimates is generally large, so the confidence intervals are frequently overlapping.

**Figure 2:**
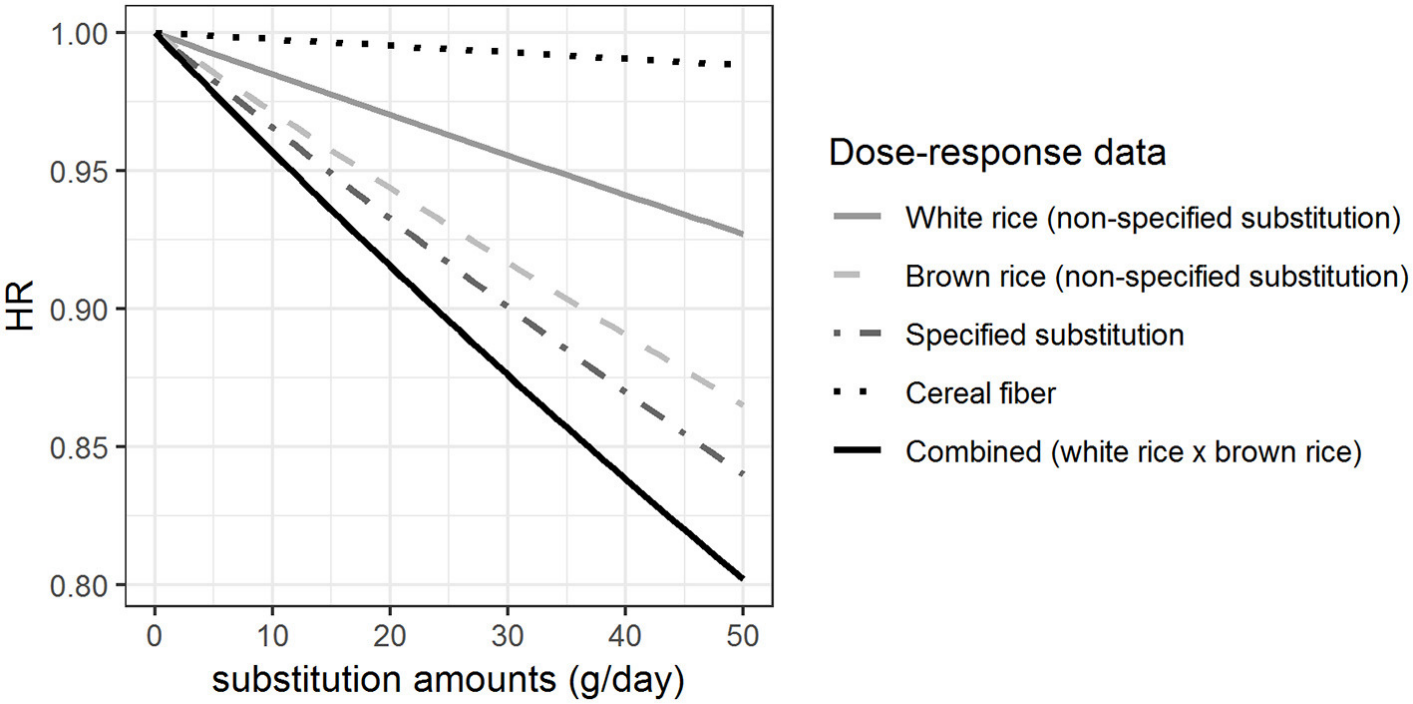
Hazard ratios (HRs) for rice substitution when different approaches are taken for the health effect characterization. The HRs for Type 2 Diabetes Mellitus of substituting up to 50 g/day of white rice by brown rice, modeled based on the β-coefficients shown in Figure 1.

Figure 2 shows the HRs calculated per gram hypothetical substitution (0–50 g/day) of white rice by brown rice based on these β-coefficients, i.e., the HRs for non-specified substitution of white rice, the non-specified substitution of brown rice, the specified substitution of white rice by brown rice, cereal fiber intake, and on the combined HRs from the dose-response functions based on the non-specified substitutions of white rice and brown rice, respectively.

We found that there were differences in the calculated HRs associated with the substitution depending on the approach taken for the health effect characterization. Our results show that there is a difference in the estimated effect sizes when using association measures describing T2DM risk due to hypothetical increased intake of cereal fiber intake (i.e., the food component) compared with using association measures for the consumption (i.e., the non-specified substitution) of white rice and brown rice, respectively (Figure 2). Furthermore, the effect size of the hypothetical substitution of white rice by brown rice estimated from the association measures for the specified substitution is different from the combined HR for the simultaneous hypothetical increase in brown rice consumption and decrease in white rice consumption (non-specified substitutions) for a given amount of rice substituted as illustrated in Figure 2. Overall, with 50 g substitution per day, depending on the approach taken for health effect characterisation, the point estimate for the HR would range from 0.80 (with combined non-specified substitutions) to 0.99 (using the food component DR). These would yield substantially different health impact estimates in an RBA.

**Figure 3:**
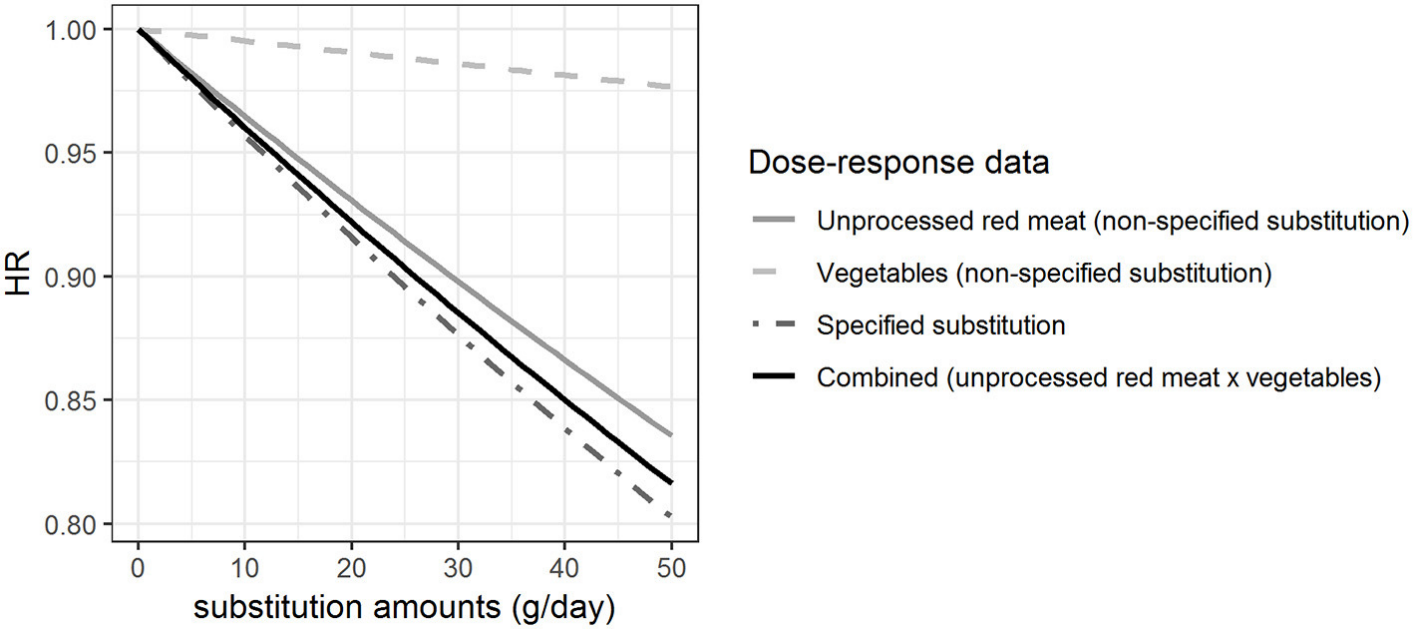
Hazard ratios (HRs) for substitution of unprocessed red meat by vegetables when different approaches are taken for the health effect characterization. The HRs for myocardial infarction of substituting up to 50 g/day of white rice by brown rice among women, modeled based on the β-coefficients shown in Figure 1.

Figure 3 shows the HRs estimated per gram hypothetical substitution of unprocessed red meat by vegetables based on the β-coefficients shown in Figure 1. HRs were estimated for the non-specified substitution of vegetables, the non-specified substitution of unprocessed red meat, the specified substitution of unprocessed red meat by vegetables, and the combined HRs from the dose-response functions derived for the non-specified substitutions of vegetables and unprocessed red meat. Our results show that the HR for the increased vegetable consumption (HR = 0.98 with 50 g substitution per day) was different from the HRs calculated for the other substitutions with HR between 0.80 and 0.84 with 50 g substitution per day. We found that there was a difference between the HRs estimated from the specified substitution of unprocessed red meat by vegetables and the combined HRs calculated from the HRs for the simultaneous increase in vegetable consumption and decrease in unprocessed red meat consumption (non-specified substitutions), although this difference is very small (Figure 3).

## Discussion

In a study of the potential differences in the estimated health impact of a dietary change, we compared the use of dose-response functions relating the change in intake of a food component, the change in intake of foods, and a substitution of foods to a health effect, based on two case studies on substituting white rice by brown rice and unprocessed red meat by vegetables. We found that RBAs potentially obtain different results depending on the approach taken for the health effect characterization.

### Comparing the Use of Association Measures for a Food Component and a Whole Food

We found that the estimated effect size for a hypothetical increased intake of cereal fiber was much lower than the effect size estimated from the dose-response functions for the non-specified substitutions of white rice and brown rice, respectively. If the assessment were based on the dose-response function for the food component instead of the food, a lower health impact would be attributed to the hypothetical change in rice consumption presented in this study. The association measure for cereal fiber intake and T2DM was derived from another study (21) than the association measures for the specified and non-specified substitutions (22). It may be unfair to compare the association measures as they are influenced by many factors, such as the differences in the populations investigated including background incidence of disease and the distribution of confounders. However, due to a lack of sufficient knowledge and data on the health impact of many foods and food components, there is usually little choice in selecting approach for health effect characterization in quantitative RBAs. Therefore, association measures from different populations may have to be used and compared in the same assessment of a given population, even though these types of bias may come along.

In addition to inter-study differences, there could be other compounds in brown rice than fiber that explain the additional beneficial effect of increased brown rice consumption compared with the corresponding increase in cereal fiber intake on T2DM risk. Moreover, it is very likely that the HR for cereal fiber refers to fibers from wheat, oats, barley or rye to a higher degree than from rice, potentially resulting in a biased estimate of the effect of fiber from rice on T2DM risk. A part of the difference may also be explained by the complex food matrix, i.e., the potential interplay between different components in food and the physical structure, which may affect digestion and absorption and accordingly influence the diet-disease association (32). The challenges in evaluating individual food components in food and their synergistic effects have been addressed by others (33–35). They emphasized the importance of conducting studies, ideally randomized controlled trials (RCT), to further investigate the association between whole foods and diseases, rather than considering food components in isolation. However, they also acknowledged the difficulty in conducting such studies.

Food processing is another factor that has an impact on the components present in food—both nutrients and contaminants. In our RBA of components of rice, we accounted for cooking of rice by accounting for the water uptake and thus the lower amount of fiber per gram of rice. Meanwhile, we did not account for any potential breakdown of fiber during cooking. Although cooking may affect the content of other nutrients, in this case the effect of food processing is expected to be negligible.

The differences in effect size illustrated in this study, comparing the use of association measures for a food component (fiber) and whole foods (white or brown rice), cannot necessarily be generalized to other component-food pairs. Thus, the application of dose-response data describing an association between a health effect and a food component compared with that of the whole food should be assessed in more case studies before any inferences can be made.

In case of an RBA at the food level, we propose to apply evidence describing associations between whole foods and a health effect, if the evidence is convincing. In that case, we account for the potential effects of the food matrix and the potential effect from other unidentified components in the food. If the evidence of a health effect is convincingly associated with the intake of a food component in the food and not with the food itself, this evidence should be used in the health impact estimation, bearing in mind that this may lead to an over- or underestimation of the health impact. Lastly, we recommend performing a scenario analysis, investigating the implications of the choice of approach for the health effect characterization on the results, when evidence of an association is available for both a food component and a food to investigate the impact of the different approaches. In the future, it should be investigated whether a clear relationship exists between food matrix and the modulation of the availability of food components to make simple assumptions about the health effect of consuming a food.

### Comparing the Use of Association Measures for a Specified and Combined, Non-specified Substitution

We compared the use of association measures derived from a specified substitution analysis and combined association measures from non-specified substitution analyses for estimating health impact of food substitutions. The effect sizes estimated in the case study on substitution of white rice by brown rice varied somewhat from each other. The results indicated that the association measure for the non-specified substitution of brown rice might already cover a substitution, likely of white rice. However, the difference in the effect size for a given substitution amount estimated from the combined HRs compared with those based on the specified substitution suggested that substitution of other foods than just rice was covered by the association measure for the non-specified substitutions of brown rice and white rice. These findings emphasized the importance of specifying substitution in the statistical analyses of observational studies.

In the example with unprocessed red meat substituted by vegetables, our findings were similar to the case study on rice; we found that other substitutions than just that between unprocessed red meat and vegetables may be covered in the association measures for the non-specified substitutions of unprocessed red meat and vegetables, and MI. In contrast to the example on rice, we found that the use of the combined HRs based on the HRs calculated from the non-specified substitutions of vegetables and unprocessed red meat, respectively, would slightly underestimate rather than overestimate the health impact of the substitution compared with the use of the HR for the specified substitution.

The fact that the association between consumption of a given food or food component and risk of disease is dependent on the replaced food or food components, has been highlighted by others (36, 37). In most observational studies, total energy intake is included in the statistical analyses in order to account for confounding and to reduce the influence of measurement error. Also, adjustment for total energy intake is important to be able to separate the specific effects of intake of foods or nutrients from the effect of energy intake. However, with total energy intake included in the model, intake of foods or nutrients is then compared with a mix of other foods or nutrients in proportion to the calories contributed. As the association between a disease and intake of a specific food or nutrient depends on the replaced food or nutrient, it would be preferable to specify the substitutions, along with the adjustment for energy intake. Such food substitutions have only been specified in few observational studies.

The use of association measures based on non-specified substitutions in quantitative RBAs results in an estimate of health impact covering an unknown substitution. However, in some situations, like the example of substituting white rice by brown rice, it seems reasonable to assume that the association measure for a non-specified substitution already covers the substitution under investigation. On the other hand, the combined HRs calculated from the non-specified substitution of unprocessed red meat and vegetables only showed a slight underestimation compared with the HRs calculated based on the specified substitution of the two foods. Thus, there is a probability of over- or under-estimating the health impact of a substitution if the combined HRs are applied to estimate the impact of the concomitant increase in consumption of one food and decrease in consumption of another food on the same health outcome.

### Implications for Quantitative RBAs of Food and Food Substitutions

The choice of approach for health effect characterization in quantitative RBA of foods may be challenging. Risk-benefit assessors may choose to use association measures describing the relationship between intake of either a food component in the given food or the whole food as such and a health effect in humans. Existing quantitative RBAs of foods have taken different approaches in calculating the health impact associated with a change in diet. The majority of RBAs has investigated the health impact of increased fish consumption using different approaches in modeling the health impact (11). Fish contain nutrients such as the omega-3 fatty acids docosahexaenoic acid (DHA) and eicosapentaenoic acid (EPA), which have been linked with both improved fetal neurodevelopment and decreased risk of fatal coronary heart disease (CHD). However, fish may also contain contaminants such as methyl mercury, which has been associated with adverse effects on fetal neurodevelopment (38). The impact of increased fish consumption on the risk of fatal CHD has been investigated in a range of RBAs. While some have linked fatal CHD risk to the intake of fish as such (20, 39), others have linked the health outcome with the intake of DHA and EPA, in fish (14, 18, 40). Using the association measure for only these specific fatty acids in the quantitative assessment may under- or over-estimate the effect of the food matrix and the whole food as such on the health outcome. Thus, these assessments may obtain different results of the impact of changes in fish consumption on risk of fatal CHD, potentially having an impact on the overall balance between risks and benefits of fish consumption. Nevertheless, the results of the studies mentioned in this specific example all pointed towards an overall beneficial effect of increased fish consumption when intake of highly contaminated fish was low (14, 18, 20, 39, 40). More studies comparing the relationship between intake of whole foods vs. food components and health effects in humans are needed to get a more comprehensive overview of the consequences of the choices made in RBA as well as in other types of health impact assessments. From the case studies, it remains unclear to what extent the difference in results depends on the data applied for the approach or the type of approach itself.

Only few RBAs have quantified the health impact of food substitutions (14, 18, 40). Both Hollander et al. (14) and Thomsen et al. (18, 40) investigated the substitution of meat by fish. However, neither of the studies based the health impact quantification on association measures for specified substitution of meat by fish. In addition, none of the health outcomes considered in the two studies were the same for both meat and fish. Although accounting for substitution in the exposure assessment, the application of association measures from non-specified substitution analyses in the quantification of health impact of increased fish and decreased meat consumption will lead to an estimate of health impact that covers other food substitutions than just that of meat by fish. Likewise, RBAs that quantify the health impact of changing the intake of only one food will cover unknown substitutions if association measures for non-specified substitutions are applied. As a result, the health impact of the theoretical interventions investigated in RBAs may be biased.

As shown in Figure 1, the CI and median value of the health impact will be different for the individual dose-response relationships. However, the figure also shows that the CIs around several of the β-coefficients overlap, which implies that they are not significantly different. Hence, in a statistical test, many of the health impacts derived from the association measures used in the current study would not be significantly different either. As quantitative RBAs usually apply the median value (with or without CI) of the effect parameters for the comparison of health impact estimates of different intake scenarios, this lack of statistical significance is not of crucial importance for RBA. Characterization of the uncertainty is part of RBA (12) and, as our study shows, this characterization will be different depending on the dose-response relationship used. Furthermore, the observed CIs stress that the uncertainty around health impact estimates is considerable, even if the underlying data is of high quality. There are also other sources of uncertainty associated with the dose-response functions applied in RBA, including that associated with the model structure. In this study, we used a rather simple log-linear model to describe the dose-response functions derived from the HRs reported in the original studies. The use of more advanced methods such as a more complex regression model could potentially provide estimates with less uncertainty of the dose-response relations. However, the approach taken in this study is an approach that is often taken in health impact assessments, including the Global Burden of Disease Study (41). Still, we emphasize the importance of accounting for and quantifying the uncertainty associated with the dose-response models used in such assessments.

Our results showed how the choice of approach for health effect characterization may have implications for the results of health impact quantified in RBA. By conducting calculations only at the level of a food component, we may ignore the potential impact of the food matrix and the whole food as such on the diet-disease association. Furthermore, calculations conducted based on dose-response functions derived from non-specified substitution analyses of observational studies cover an underlying substitution. It may not be straightforward to identify which substitution is covered if not specified in the statistical analysis. Indeed, for RBAs accounting for substitution of foods, there is a possibility that association measures based on non-specified substitutions cover another substitution than what is investigated in the RBA. While we acknowledge that limited data for health effects related to substitution of foods is available from observational studies, we emphasize the need for scrutinizing the underlying assumptions and the limitations of the data used.

## Conclusions

We investigated the relative differences in effect size of dietary changes when taking different approaches for health effect characterization in RBA. We explored and compared the use of association measures derived from observational studies linking disease risk with a food component, a non-specified substitution of foods, or a specified substitution of foods. We found that results may vary depending on the approach taken for the health effect characterization. In future RBAs, similar scenario analyses are encouraged when multiple options for health effect characterization are possible. The underlying assumptions and limitations of the dose-response relations used should be acknowledged and communicated along with the final results to provide transparency of the choices made. Finally, to ensure better data for quantitative RBA of foods, specification of food substitution in observational studies is warranted.

## Data Availability Statement

The original contributions presented in the study are included in the article/Supplementary Material, further inquiries can be directed to the corresponding author.

## Author Contributions

ST and MN developed the research idea. ST, MN, RA, LJ, MP, and MJ contributed to the framing of the study. MJ, HM, MO, GR-H, and RA searched and reviewed the literature. ST conducted the calculations and drafted the manuscript together with MN. All authors contributed to the interpretation and discussion of results and reviewing the manuscript. All authors approved the final manuscript.

## Conflict of Interest

The authors declare that the research was conducted in the absence of any commercial or financial relationships that could be construed as a potential conflict of interest.
