# Supplementary material for: Approaches for Health Effect Characterization in Risk-Benefit Assessment of Foods: A Comparative Case Study
Source: Front Nutr. 2021 Jul 9;8:607929. doi: 10.3389/fnut.2021.607929 (PMC8298756; doi:10.3389/fnut.2021.607929)
Supplement: Supplementary file 1 [file Table_1.docx]

# Supplementary Material

## Estimation of beta-parameters for exponential dose-response function

The beta parameters were estimated in terms of per gram substitution. As an example, we will show how the beta parameters were calculated in the case study on substituting white rice by brown rice and the risk of type 2 diabetes mellitus (T2DM).

The beta parameters for the non-specified substitution of white rice and brown rice, respectively, were estimated by fitting an exponential function to the data reported by (22), given in Table 1, by translating one serving to 150 g of cooked rice as reported in (22). By doing this, we obtained a beta parameter of $\beta_{white}=0.0015$ and $\beta_{brown}=-0.0029$ expressed in terms of per g increase in consumption of white rice and brown rice, respectively. These were converted in order to be expressed in terms of g substitution of white rice (decreased consumption) by brown rice (increased consumption). For brown rice, the beta parameter was the same when expressed in terms of per g increase as in per g substitution of white rice by brown rice. For white rice, the beta parameter has an opposite sign when expressed in terms of per gram substitution of white rice by brown rice, as the white rice consumption decreases due to the substitution, so $\beta_{white,sub}=-0.0015$ and $\beta_{brown,sub}=-0.0029$.

For the specified substitution of white rice by brown rice, (22) reported a HR for T2DM of 0.84 per 50 g substitution per day. The beta parameter for the specified substitution of white rice by brown rice was derived by using equation (1), i.e.:

$$\ln\left( RR \right)=\beta_{sub}x=>\ln\left( 0.84 \right)=\beta_{sub}\cdot50$$

This leads to:

$$\beta_{sub}=\frac{ln(0.84)}{50}=-0.0035$$

We also estimated a combined beta parameter for the substitution of white rice by brown rice based on the beta parameters estimated from the HRs for the non-specified substitution of white rice and brown rice, respectively, reported in (22) Sun et al. (2010). This approach may be necessary in RBAs when HRs for the specified substitution have not been derived. The beta parameter for the combined dose-response function, expressed in terms of per g substitution of white rice by brown rice, was found by taking the difference between the beta parameters estimated for the non-specified substitution of brown rice and white rice, respectively, expressed in per g increased consumption, which is:

$$\beta_{combined, sub}=\beta_{brown}-\beta_{white}=-0.0029-0.0015=-0.0044$$

The beta parameter for the association between cereal fiber intake and T2DM was derived based on the HR for T2DM reported by (21) of 0.75 per 10 g cereal fiber per day. I.e.

$$\ln\left( RR \right)=\beta_{fiber}x=>\ln\left( 0.75 \right)=\beta_{fiber}\cdot10$$

This leads us to:

$$\beta_{fiber}=\frac{ln(0.75)}{10}=-0.029$$

The beta parameter is expressed in per g increase in cereal fiber intake. However, we wanted the beta parameter to be expressed in terms of per g substitution of white rice by brown rice. In order to do this, we used information on the amount of fiber in (uncooked) white and brown rice and combined this with information on the change in mass of white and brown rice upon cooking. The fiber content in uncooked white and brown rice were obtained from the Danish Food Composition Databank (29) and conversion factors used to convert uncooked rice into cooked rice were obtained from (41). These numbers are given in Table S1. The fiber content in the cooked white and brown rice was calculated by dividing the fiber content (in g/g) in white rice and brown rice by the conversion factor for white rice and brown rice, respectively. These numbers are given in Table S1.

**Table S1 Parameters used to calculate changes in fiber intake due to substitution of cooked white rice by cooked brown rice.**

|  | **White rice** | **Brown rice** |
| --- | --- | --- |
| **Conversion factor** | 2.5 | 3.8 |
| **Fiber content in uncooked rice (g/g)** | 0.0070 | 0.042 |
| **Fiber content in cooked rice (g/g)** | 0.0028 | 0.011 |

Based on the calculated fiber content in the cooked white rice and brown rice, we could calculate the difference in fiber intake (g) per g substitution by taking the difference in fiber content in cooked brown rice and cooked white rice. We calculated a difference/increase *z =* 0.0083 g of fiber per g substitution of white rice by brown rice based on the numbers given in Table S1. This difference was then multiplied with the beta parameter estimated for a g increase in fiber intake:

$$\beta_{fiber,sub}=\beta_{fiber}\cdot z= -0.029\cdot0.0083=-0,00024$$

The confidence intervals around the beta parameters were derived in a similar way, based on the reported confidence intervals of the HRs.

## Calculation of hazard ratios (HRs) for specific substitution amounts

The estimated Beta-parameters were then used to describe exponential dose-response functions, describing the relation between white rice, brown rice, substitution of white rice by brown rice, and increased fiber intake due to substitution of white rice by brown rice. All dose-response functions were expressed in terms of per g substitution as the Beta-parameters. The dose-response functions were then combined with hypothetical amounts of white rice substituted by brown rice to explore relative differences in the estimation of risk of T2DM when either of the approaches for health effect characterization were used. Here we show some examples of how we calculated the HRs for a given amount of hypothetical substitution of white rice by brown rice. We used the Beta-parameters derived in the previous section of this Supplementary Material (see also Table S1). The HRs were then estimated as the ratio between the HR at a given amount of substitution relative to a zero substitution.

Example 1 – hazard ratio (HR) for increased brown rice consumption due to a substitution $y_{sub}=5g$ compared to no substitution, $y_{ref}=0g$:

$${HR}_{brown,sub}=\frac{e^{\beta_{brown,sub\cdot y_{sub}}}}{e^{\beta_{brown,sub\cdot y_{ref}}}}=\frac{e^{-0.0029\cdot5}}{e^{-0.0029\cdot0}}=0.986$$

Example 2 – HR for decreased white rice consumption due to a 5 gram substitution $y_{sub}=5g$ compared to no substitution, $y_{ref}=0g$:

$${HR}_{white,sub}=\frac{e^{-0.0015\cdot5}}{e^{-0.0015\cdot0}}=0.992$$

The HRs calculated for each of the approaches for health effect characterization (white rice, brown rice, combined approach, specified substitution, cereal fiber) for a 0-5 g/d substitution are listed in Table S2.

**Table S2 Hazard ratios calculated based on dose-response functions derived per g substitution of white rice by brown rice**

| Substitution of cooked white rice by cooked brown rice | Hazard ratios calculated from dose-response relationship | | | | |
| --- | --- | --- | --- | --- | --- |
| g/day | White rice | Brown rice | Substitution | Combined | Cereal fiber |
| 0 | 1 | 1 | 1 | 1 | 1 |
| 1 | 0.998 | 0.997 | 0.997 | 0.996 | 1 |
| 2 | 0.997 | 0.994 | 0.993 | 0.991 | 1 |
| 3 | 0.995 | 0.991 | 0.990 | 0.987 | 0.999 |
| 4 | 0.994 | 0.988 | 0.986 | 0.983 | 0.999 |
| 5 | 0.992 | 0.986 | 0.983 | 0.978 | 0.999 |
